# The protective role of α-lipoic acid in hypothyroidism-related ovarian damage

**DOI:** 10.1007/s00210-026-05163-6

**Published:** 2026-03-06

**Authors:** Ahmet Türk, Muhittin Önderci, Seda Koçak, Gülşen Bayrak, Zehra Demiray Asoglu

**Affiliations:** 1 Adiyaman University Faculty of Medicine Department of Histology-Embryology, Adiyaman, Turkey; 2 Adiyaman University Faculty of Medicine Department of Biochemistry, Adiyaman, Turkey; 3 Kırşehir Ahi Evran University Faculty of Medicine Department of Physiology, Kırşehir, Turkey; 4 Uşak University Faculty of Medicine Department of Histology-Embryology, Uşak, Turkey; 5 Hatay Mustafa Kemal University Faculty of Medicine Department of Histology-Embryology, Hatay, Turkey

**Keywords:** Hypothyroidism, Alpha-Lipoic acid, Ovarian damage, PI3K/AKT, GRP78

## Abstract

This study aimed to investigate the potential protective effects of α-lipoic acid on ovarian tissue damage provoked by propylthiouracil (PTU)-induced hypothyroidism in rats, focusing on oxidative stress, hormonal changes, and Phosphoinositide 3-kinase / Protein kinase B (PI3K/AKT) signaling pathway activity. Twenty-eight female Sprague–Dawley rats (10–12 weeks, 340–360 g) were randomly divided into four groups (*n* = 7 each): Control, Hp, Hp + α-lipoic acid, and α-lipoic acid. Hypothyroidism was induced by adding 0.05% PTU to drinking water for 8 weeks. α-lipoic acid (100 mg/kg/day) was administered orally. At the end of the experiment, serum and ovarian tissues were collected for biochemical, histological, and immunohistochemical analyses. Serum thyroid hormones, gonadotropins, TAS, TOS, GRP78, PI3K, and AKT1 levels were measured. Ovarian follicle counts, histopathological changes, and immunohistochemical expression of GRP78, PI3K, and AKT1 were evaluated. PTU administration significantly decreased serum T3, T4, TAS, LH, PI3K, and AKT1 levels while increasing TSH, FSH, TOS, and GRP78 levels compared to the control group (*p* < 0.001). Histological analysis revealed follicular atresia, granulosa cell degeneration, inflammation, and edema in the Hp group. In contrast, α-lipoic acid treatment attenuated oxidative stress, improved folliculogenesis, reduced histopathological damage, and reversed PTU-induced hypothyroidism alterations in PI3K/AKT1 signaling and GRP78 expression (*p* < 0.05). α-lipoic acid exerts protective effects against PTU-induced hypothyroidism-related ovarian damage through its antioxidative properties and regulation of PI3K/AKT signaling. These findings suggest that α-lipoic acid may serve as a potential therapeutic agent in preserving ovarian function under hypothyroid conditions.

**Graphical Abstract:**

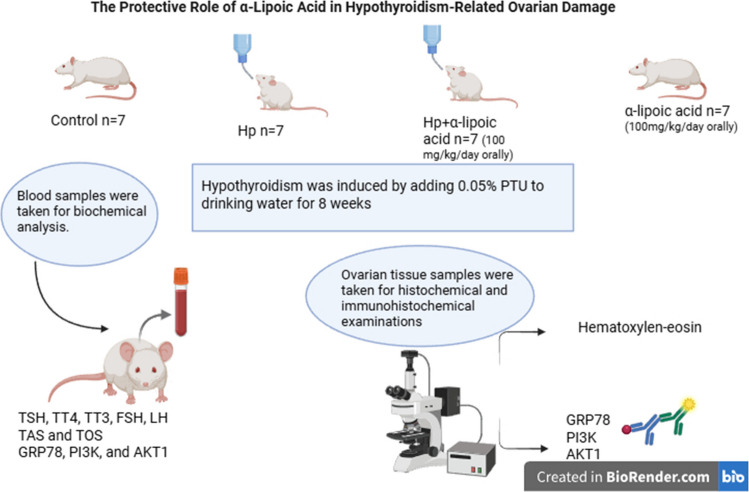

## Introductıon

Hypothyroidism is one of the most common diseases worldwide, affecting 5% of the general population (Chiovato et al. 2019). Thyroid dysfunction is one of the endocrine disorders affecting women of reproductive age (Guglielmi et al. 2018). It can cause infertility, hypo/hypermenorrhea, amenorrhea, oligomenorrhea, and recurrent pregnancy loss in women of reproductive age (Koyyada and Orsu 2020). Studies have also reported that hypothyroidism leads to premature ovarian failure, tubal obstruction, and ovulation disorders (Krassas and Markou 2019).

It is known that thyroid hormones play a role in activating the mechanism of follicular development (Silva et al. 2018). Granulosa cells (GCs) play an important role in follicular development and are responsible for converting androgens to estrogens, synthesizing progesterone, and secreting insulin-like growth factor (IGF) (Cavalcanti et al. 2023; Hayes et al. 2024) However, thyroid hormones inhibit pro-apoptotic molecules such as BAX and Caspase-3 while maintaining activation of the Phosphoinositide 3-kinase/Protein kinase B (PI3K/AKT) pathway in granulosa cells (Di Paolo et al. 2020).

Phosphatidylinositol 3-kinases (PI3Ks) are a family of enzymes that share the function of phosphorylating the 3-hydroxyl group of phosphoinositides (Rathinaswamy and Burke 2020). PI3K signaling is a fundamental pathway in regulating cell proliferation, survival, migration, and metabolism in various physiological and pathological processes. Recent studies in humans and mice have confirmed that PI3K/AKT signaling plays an important role in regulating GC growth and apoptosis during follicular development (Sun et al. 2015). Serine/threonine-specific protein kinase B (PKB), also known as AKT, is an important factor in the PI3K/AKT signaling pathway that regulates cell survival, metabolism, and proliferation, and controls cytokinesis (Kalous et al. 2023). It has been determined that AKT activation increases with short-term exposure to endoplasmic reticulum (ER) stress but decreases with long-term ER stress, and that AKT inactivation plays an important role in the induction of growth arrest and DNA damage-inducible protein 153 (GADD153) (Almanza et al. 2019). In short, the PI3K/AKT signaling pathway regulates cell growth and proliferation. Oxidative stress in GC often causes downregulation of the PI3K/AKT signaling pathway, which can disrupt the growth and proliferation of these cells, affecting ovarian function and potentially leading to various diseases (Liu et al. 2023) Glucose-regulated protein 78 (GRP78) is a well-characterized endoplasmic reticulum stress protein associated with apoptosis in the ovaries (Wang et al. 2020). Thyroid hormone and GRP78 affect fundamental functions such as growth, development, basal metabolic rate, energy production, and carbohydrate and lipid metabolism (Cicatiello et al. 2018). GRP78 induction plays important roles in maintaining ER homeostasis and protecting cells from ER stress-induced apoptosis (Lee 2025). GRP78 has been identified as a direct target of the PI3K/AKT signaling pathway, and PI3K/AKT has been shown to prevent ER stress-induced cell death (He et al. 2025).

Alpha-lipoic acid (ALA; 1,2-dithiolane-3-pentanoic acid) is a key mitochondrial cofactor with antioxidant, antimicrobial, antidiabetic, neuroprotective, and cardioprotective effects. (Khan et al. 2022). The scientific community has shown growing interest in natural medicines such as phytochemicals and Chinese herbal plants despite advances in modern medicine, driven by their unique chemical biodiversity, novel mechanisms of action, strong biological modulation capacity, high efficacy, health benefits, and relatively few side effects (Yuan et al. 2016; Morsi et al. 2024; Shawky et al. 2024). The PI3K/Akt pathway plays an important role in reproductive physiology, from oocyte meiosis maturation, acquisition of developmental capacity, and early stages of embryo development to follicle formation and activation, and ultimately ovulation (Kalous et al. 2023). Studies on the interaction of the PI3K/Akt pathway with cellular mechanisms via agents such as ALA are limited. Plasma and human cell ALA levels are insufficient to meet biological requirements, so ALA is administered as supplementation under physiological and pathophysiological conditions (Yunusoğlu and Koyuncu 2025). Understanding how ALA interacts with cellular targets and signaling pathways in ovarian tissues under pathophysiological conditions will shed light on future studies (Di Nicuolo et al. 2021).

Therefore, the present study was designed to investigate the potential protective effects of ALA on ovarian damage by propylthiouracil (PTU)-induced hypothyroidism in rats. Specifically, we aimed to evaluate its impact on thyroid hormones, gonadotropins, oxidative stress parameters (TAS, TOS), ER stress marker GRP78, and PI3K/AKT signaling both at the serum and tissue levels. In addition, histological and immunohistochemical analyses were performed to assess folliculogenesis and ovarian morphology, providing a comprehensive understanding of the therapeutic role of ALA in hypothyroidism-related ovarian dysfunction.

## Material and methods

### Chemicals and reagents

In this study, propylthiouracil (PTU; ≥ 99% purity; CAS No. 51-52-5; Sigma–Merck Aldrich, Germany) and α-lipoic acid (≥ 99% purity; CAS No. 1077-28-7; Sigma–Merck Aldrich, Germany) were used as the experimental agents. Anesthesia at the end of the experimental period was achieved using xylazine (Alfazine; Alfasan IBV, Woerden, The Netherlands) and ketamine hydrochloride (Alfamine; Alfasan IBV, Woerden, The Netherlands). Alpha-lipoic acid was prepared for administration at a dose of 100 mg/kg/day. First, the appropriate amount of alpha-lipoic acid was suspended in sterile saline and centrifuged to obtain a homogeneous mixture. The suspension was then completely dissolved in sterile saline, and the pH of the resulting solution was adjusted to 7.4 using both 1 M NaOH and 1 M HCl. The prepared alpha-lipoic acid solution was administered to the experimental groups via oral gavage (Özel et al. 2020).

### Procurement of experimental animals

The experimental animals were obtained from the Experimental Animals Application and Research Center of Adıyaman University, and all experimental procedures were conducted there. Accordingly, 28 female Sprague–Dawley rats, aged 10–12 weeks and weighing between 340–360 g, all in the same estrous cycle (Samples were collected using the vaginal cytology method, examined microscopically, and documented by photomicrography) were randomly divided into four groups with seven animals in each group. The rats were housed under controlled conditions with a room temperature of 22 ± 2 °C, a 12-h light/12-h dark cycle, and free access to food and water throughout a one-week adaptation period and an eight-week experimental period (Özel et al. 2020).

#### Vaginal cytology

During the collection of vaginal smear samples, rats were gently grasped by the tail and tail root and held in an upside-down position. Under sterile conditions, a sterile swab moistened with physiological saline was inserted into the vagina and rotated fully to collect cellular material from the vaginal lumen and walls. The cells on the swab were spread homogeneously onto clean, pre-labeled slides. The cells obtained from the vaginal region were air-dried to fix them onto the slide.The prepared slides were completely covered with a 1% fuchsin solution using a dropper and stained, then briefly washed under running water to remove excess dye. After the staining process was completed, the vaginal smear preparations were air-dried and evaluated under a light microscope (Leica DM500, Germany) without using a coverslip. Cellular structures belonging to different stages of the estrus cycle were photographed using a camera-equipped light microscope (Leica DM500, Germany).

#### Formation of experimental groups

##### Group 1 (Control Group) (n:7)

No procedures were performed on any rats during the 8-week experimental period.

##### Group 2 (Hp Group) (n:7)

PTU was added to the rats' drinking water at a concentration of 0.05% (Panda et al. 2021). Drinking water was replaced every other day for 8 weeks, and PTU was prepared and added as the water ran out.

##### Group 3 (Hp + α-lipoic acid Group) (n:7)

PTU was added to the drinking water of the rats at a concentration of 0.05% for 8 weeks (Panda et al. 2021) and at the same time, 100 mg/kg body weight α-lipoic acid was administered orally for 8 weeks (Oraebosi et al. 2022).

##### Group 4 (α-lipoic acid group) (n:7)

100 mg/kg body weight α-lipoic acid was administered orally for 8 weeks (Oraebosi et al. 2022).

#### Collection of tissue and serum samples

At the end of the experiment, all groups were anesthetized with xylazine (Alfazine; Alfasan IBV, Woerden, The Netherlands) and ketamine (50 mg/kg, Alfamine; Alfasan IBV, Woerden, The Netherlands). Blood samples were collected intracardially, ovaries were removed, and the rats were subsequently decapitated to terminate the experiment. Ovarian tissue samples were washed with physiological saline and then placed in 10% neutral formaldehyde for storage at room temperature for histological studies. Blood was placed in serum separation tubes for biochemical analysis; and after clotting, serum samples were centrifuged at 1107 G for 10 min (Hettich ROTINA 380/380R, Germany) and stored at −80 °C (Nüve DF-270, Turkey) until the day of analysis.

### Biochemical analyses

#### Analysis of TSH, TT4, TT3, FSH, LH levels in serum samples

Analysis of Thyroid Stimulating Hormone (TSH) (IU/mL), Total Thyroxine (T4) (ng/dL), Total Triiodothyronine (T3) (pg/mL), Follicle Stimulating Hormone (FSH) (mIU/mL), Luteinizing Hormone (LH) (mIU/mL) parameters in the serum of the collected blood samples were measured using the chemiluminescence method on a Siemens brand, Advia Centaur XP model autoanalyzer.

#### Analysis of TAS and TOS levels in serum samples

Total antioxidant status (TAS) (Elabscience, Cat no: E-BC-K801-M) and total oxidant status (TOS) (Elabscience Cat no: E-BC-K802-M) were measured in serum samples using the ELISA method. Serum levels were measured according to the procedures specified in the Elabscience brand kit catalog. An automatic washer Bio-Tek ELX50 (BioTek Instruments) was used for plate washing, and ChroMate, Microplate Reader P4300 devices (AwarenessTechnology Instruments) were used for absorbance readings. The unit of measurement for the test results was mmol Trolox and H₂O₂ equivalent/L for TAS and μmol H₂O₂ eq/L for TOS in serum samples.

#### Analysis of GRP78, PI3K, and AKT1 levels in serum samples

The The enzyme-linked immunosorbent assay (ELISA) method was used to determine the levels of GRP78 (Fine test Code no: ER0562), AKT1 (RAC-alpha serine/threonine-protein kinase) (Fine test Code no: ER1268), and PI3K (Fine test Code no: ER1910) in serum samples. Serum levels were measured according to the manufacturer’s instructions. An automatic washer Bio-Tek ELX50 (BioTek Instruments) was used for plate washing, and ChroMate, Microplate Reader P4300 devices (AwarenessTechnology Instruments) were used for absorbance readings. The units of the test results were ng/mL for GRP78, ng/mL for AKT1, and pg/mL for PI3K in serum samples.

### Histological analyses

Tissue samples obtained from the sacrificed animals were fixed in 10% neutral formaldehyde solution for one week, followed by routine tissue processing for light microscopic analysis. Routine histological procedures (consisting of alcohol, xylene, and paraffin series) were performed using an automated tissue processor (Leica TP1020, Nussloch, Germany). From the prepared paraffin blocks, 5 µm-thick sections were cut using a Thermo Shandon Finesse ME microtome (Thermo Fisher Scientific, Cheshire, UK). The sections were stained with hematoxylin and eosin (H&E) to evaluate follicle and corpus luteum counts as well as the presence of tissue damage among the groups, and then examined and photographed under a light microscope (Leica DM 500).

#### Semi-quantitative scoring of follicle counts

Serial paraffin sections of 5 µm thickness were stained with hematoxylin and eosin. For microscopic examination, sections were selected using a systematic random sampling method (10% sampling). Follicles and corpora lutea were counted in every 10th section of the ovaries, with each examined section being approximately 50–60 µm apart from the next (Koçak et al. 2025). Follicles were classified according to the following definitions: Primordial follicle: surrounded by a thin, single layer of follicular epithelial cells. Primary follicle: surrounded by a single or multiple layers of follicular epithelium, which changes from iso-prismatic to high prismatic cells. Secondary follicle: characterized by more than two layers of granulosa cells surrounding the oocyte and the initiation of antral cavity formation. Graafian (tertiary) follicle: identified by a single large antrum filled with follicular fluid, lined by granulosa cells, with the oocyte surrounded by cumulus cells. Atretic follicle: degenerative follicle characterized by pyknosis of granulosa cells, degeneration of the zona pellucida or oocyte, and the presence of granulosa cell debris in the antral cavity. All sections were evaluated and photographed using a light microscope (Leica DM 500) (Furat Rencber et al. 2018).

#### Histological evaluation of tissue damage using hematoxylin & eosin staining

Ovarian tissues fixed in 10% formaldehyde were processed with routine histological procedures and embedded in paraffin blocks. Sections of 5 μm thickness were selected by systematic random sampling, stained with hematoxylin and eosin (H&E), and examined under a Leica DM500 microscope. Semi-quantitative analysis was performed, and representative images were photographed. Based on previous studies, histological parameters evaluated in ovarian tissue included hemorrhage, follicular cell degeneration, inflammatory cell infiltration, and edema. Tissue damage was graded as follows: 0 = no damage; 1 = mild (< 25%); 2 = moderate (26–50%); 3 = severe (> 51%). The mean total scores were calculated separately for each group (Bancroft and Layton 2018; Kaplan and Türk 2022).

#### Immunohistochemical analysis of GRP78, PI3K, and AKT1 expression

Sections obtained from paraffin blocks and mounted on poly-L-lysine-coated slides were deparaffinized and rehydrated through a graded alcohol series, followed by heat-induced antigen retrieval in citrate buffer solution (pH 6) using a microwave oven (750 W) for 12 min. After washing with phosphate-buffered saline (PBS), the sections were incubated with hydrogen peroxide solution for 6 min to block endogenous peroxidase activity, followed by PBS rinses (3 × 5 min). A blocking solution (TA-125-UB) was applied for 5 min, after which sections were incubated for 60 min at room temperature with the following primary antibodies: GRP78 (1:100; Affinity Biosciences, cat no: AF5366 China), AKT1 (1:100; Abcam, cat no: AF6261, China), and PI3K (1:100; Affinity Biosciences, cat no: AF6241, China). After washing, sections were incubated with a secondary antibody (TA-125-SB) for 30 min in a humid chamber, followed by incubation with streptavidin-peroxidase (TS-125-HR, Lab Vision Corporation, USA) for 30 min at room temperature. Immunoreactivity was visualized with 3-amino-9-ethylcarbazole (AEC) chromogen, and counterstaining was performed with Mayer’s hematoxylin. Slides were washed with phospate buffer saline (PBS) and distilled water, mounted, and examined under a Leica DM500 microscope. Positive control tissues recommended in the manufacturer’s datasheets for each antibody were used to verify staining specificity. Negative control sections were processed by omitting the primary antibody while keeping all other steps identical. Immunoreactivity was scored based on the extent (0.1: < 25%; 0.4: 26–50%; 0.6: 51–75%; 0.9: 76–100%) and intensity (0: none, + 0.5: very weak, + 1: weak, + 2: moderate, + 3: strong). Histoscore was calculated using the formula: Histoscore = extent × intensity (Kiernan 2015; Turk et al. 2025).

### Statistical analysis

The data obtained from the study were analyzed using SPSS 22.0 (SPSS, Chicago, IL, USA) statistical software. Normality of distribution was assessed by the Kolmogorov–Smirnov test. Data with normal distribution were expressed as mean ± standard deviation (SD) and analyzed by one-way analysis of variance (ANOVA), followed by Tukey’s post hoc test for subgroup comparisons when ANOVA indicated statistical significance. For data not following a normal distribution, the Kruskal–Wallis test was used, followed by Dunn’s post hoc test for multiple comparisons. A p-value < 0.05 was considered statistically significant for all analyses. Graphical representations of the collected data were created using GraphPad Prism 8.4 (GraphPad Software, San Diego, CA, USA).

## Results

### Biochemical analysis results

#### Serum TSH, T4, T3, FSH, LH, TAS, TOS, GRP78, PI3K, and AKT1 levels

**Fig. 1:**
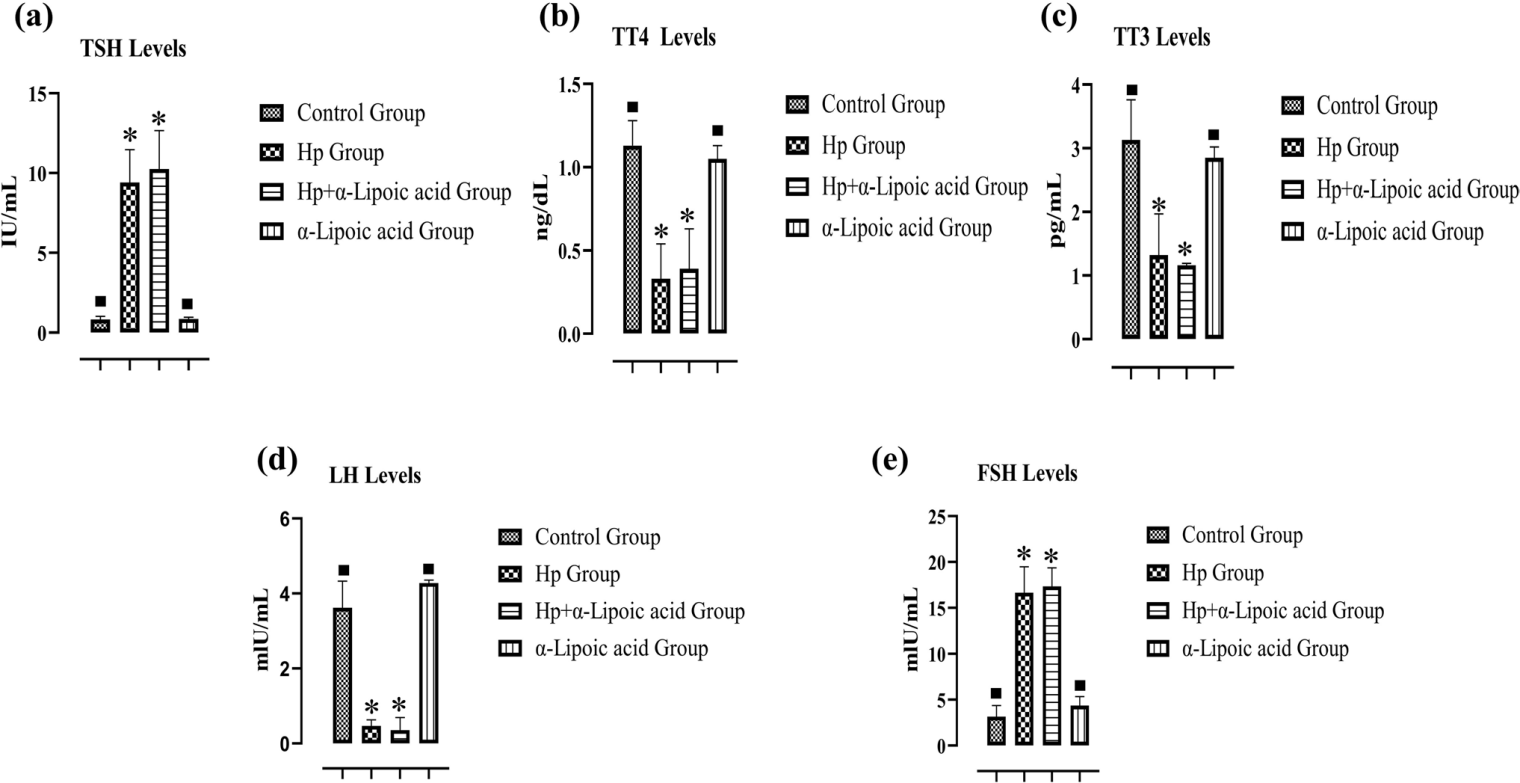
TSH, T4, T3, LH, and FSH levels are observed in rat serum samples from all groups. Data were analyzed using one-way ANOVA followed by Tukey’s post hoc test. (▪ Compared to the control group, *p* < 0.001; * compared to the Hp group, *p* < 0.001)

**Fig. 2:**
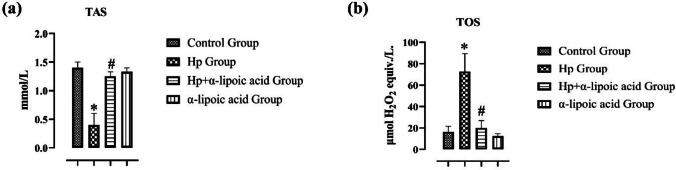
Serum TAS and TOS levels in hypothyroidism following α-lipoic acid administration. Data were analyzed using one-way ANOVA followed by Tukey’s post hoc test (* Compared to the control group (*p* < 0.001), # Compared to the Hp group (*p* < 0.001))

**Fig. 3:**
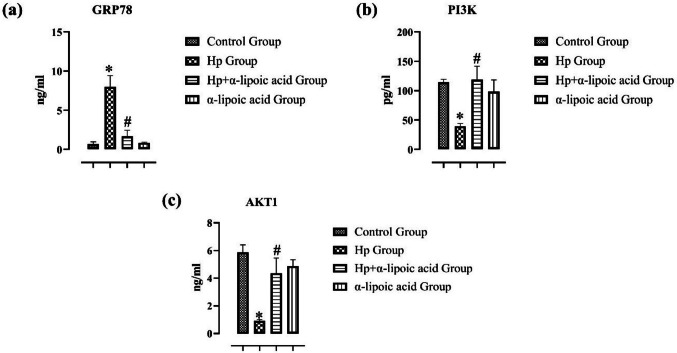
GRP78, PI3K, and AKT1 levels in rat serum samples from all groups. Data were analyzed using one-way ANOVA followed by Tukey’s post hoc test (* compared to the control group, *p* < 0.001; # compared to the Hp group, *p* < 0.001)

TSH levels were similar in the control and α-lipoic acid groups (*p* = 0.745). Compared with the control group, TSH levels were significantly increased in both the Hp group and the Hp + α-lipoic acid group (*p* < 0.001). However, no significant difference was observed between the Hp and Hp + α-lipoic acid groups (*p* = 0.537) (Fig. 1). T4 levels were similar between the control and α-lipoic acid groups (*p* = 0.581). Compared with the control group, T4 levels were significantly decreased in the Hp and Hp + α-lipoic acid groups (*p* < 0.001). No significant difference was observed between the Hp and Hp + α-lipoic acid groups (*p* = 0.721) (Fig. 2). T3 levels were similar between the control and α-lipoic acid groups (*p* = 0.626). Compared with the control group, T3 levels were significantly decreased in the Hp and Hp + α-lipoic acid groups (*p* < 0.001). No significant difference was observed between the Hp and Hp + α-lipoic acid groups (*p* = 0.623) (Fig. 1). FSH levels were comparable between the control and α-lipoic acid groups (*p* = 0.172). Compared with the control group, FSH levels were significantly increased in the Hp and Hp + α-lipoic acid groups (*p* < 0.001). However, no significant difference was observed between the Hp and Hp + α-lipoic acid groups (*p* = 0.673) (Fig. 1). LH levels were similar between the control and α-lipoic acid groups (*p* = 0.672). Compared with the control group, LH levels were significantly decreased in the Hp and Hp + α-lipoic acid groups (*p* < 0.001). No significant difference was observed between the Hp and Hp + α-lipoic acid groups (*p* = 0.603) (Fig. 1). TAS levels were similar between the control and α-lipoic acid groups (*p* = 0.494). Compared with the control group, serum TAS levels were significantly decreased only in the Hp group (*p* < 0.001). In the Hp + α-lipoic acid group, TAS levels were significantly higher than those in the Hp group (*p* < 0.05) (Fig. 2). TOS levels were comparable between the control and α-lipoic acid groups (*p* = 0.131). Compared with the control group, serum TOS levels were significantly increased in the Hp group (*p* < 0.001). In the Hp + α-lipoic acid group, TOS levels were significantly lower compared with the Hp group (*p* < 0.05) (Fig. 2). Serum GRP78, PI3K, and AKT1 levels were measured spectrophotometrically and compared among groups. GRP78 (*p* = 0.673), PI3K (*p* = 0.516), and AKT1 (*p* = 0.322) levels were similar in the control and α-lipoic acid groups. Compared with the control group, GRP78 levels were significantly increased in the Hp group (*p* < 0.001), while both PI3K and AKT1 levels were significantly decreased (*p* < 0.001). In the Hp + α-lipoic acid group, GRP78 levels were significantly reduced (*p* < 0.001), whereas PI3K (*p* < 0.001) and AKT1 (*p* < 0.001) levels were significantly increased compared with the Hp group (Fig. 3).

### Histological analyses

#### Semi-quantitative follicle count results

Semi-quantitative analysis of different follicle types in rat ovarian tissues showed that in the control and α-lipoic acid groups, primordial, primary, secondary, tertiary, atretic follicles, and corpus luteum counts were not significantly different (*p* = 0.614, *p* = 0.909, *p* = 0.922, *p* = 0.108, *p* = 0.757, and *p* = 0.768, respectively). Compared with the control group, the HP group showed a significant reduction in healthy follicles, including primordial, primary, secondary, and tertiary follicles, as well as corpus luteum (*p* < 0.05), whereas the number of atretic follicles was significantly increased. When the Hp group was compared with the Hp + α-lipoic acid group, primordial, secondary, and Graafian follicle counts were significantly increased, while atretic follicle counts were significantly decreased (*p* < 0.05). Although the corpus luteum count was higher in the Hp + α-lipoic acid group compared with the Hp group, the difference was not statistically significant (*p* = 0.670) (Table 1).

**Table 1:** Semi-quantitative scoring results for follicle counts in rat ovarian tissues from the experimental groups. Values are given as median (min–max). Statistical analysis was performed using the Kruskal–Wallis test followed by Dunn’s post hoc test. a: Compared to the control group, *p* < 0.05; b: Compared to the Hp group, *p* < 0.05; c: Compared to the Hp + α-lipoic acid group, *p* < 0.05

Groups	Primordial follicle Median (min–max)	Primary follicle Median (min–max)	Secondary follicle Median (min–max)	Tertiary follicle Median (min–max)	Atretic follicle Median (min–max)	Corpus luteum Median (min–max)	*p* values
Control Group	61.00 (45.00–70.00)^a^	32.00 (25.00–40.00)^a^	12.00 (11.00–20.00)^a^	6.00 (5.00–8.00)^a^	1.00 (1.00–2.00)^a^	10.00 (9.00–13.00)^a^	*P* < 0,05
Hp Group	18.00 (13.00–22.00)^b^	16.00 (10.00–20.00)^b^	20.00 (16.00–26.00)^b^	2.00 (2.00–3.00)^a^	6.00 (4.00–7.00)^b^	2.00 (1.00–10.00)^b^	
Hp + α-Lipoic acid Group	47.00 (42.00–52.00)^c^	2.00 (14.00–30.00)^b^	12.00 (8.00–17.00)^c^	5.00 (4.00–6.00)^b^	2.00 (1.00–3.00)^c^	7.00 (1.00–9.00)^b^	
α-Lipoic acid Group	63.00 (48.00–72.00)^a^	31.00 (24.00–46.00)^a^	13.00 (9.00–16.00)^a^	8.00 (7.00–9.00)^a^	1.00 (1.00–2.00)^a^	10.00 (9.00–12.00)^a^	

#### Oestrous cycle determination and evaluation of ovarian damage

**Fig. 4:**
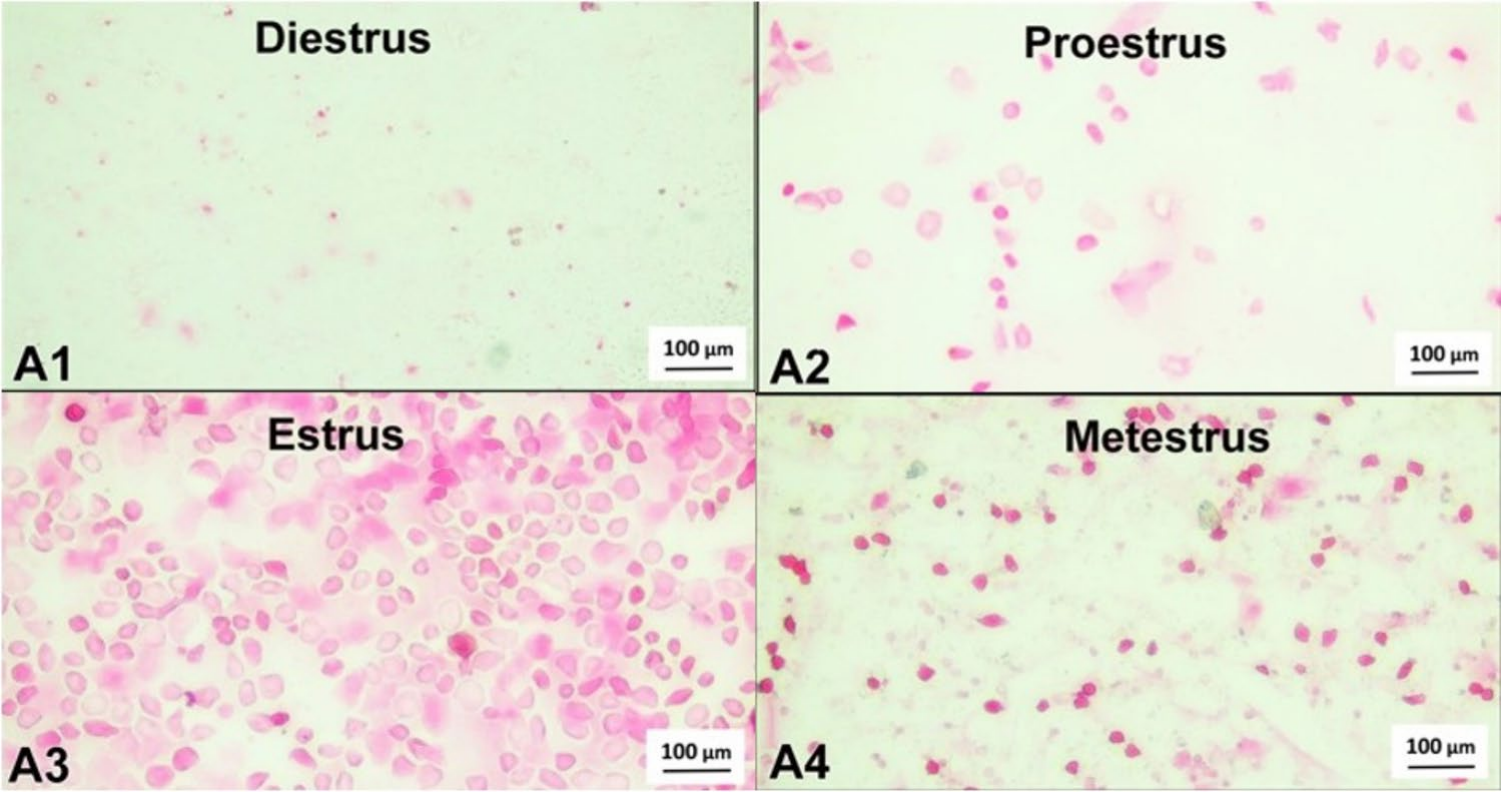
Vaginal smear cytology of rats in all groups stained with 1% aqueous fuchsin; scale bar: 100 µm A1: Diestrus A2: Proestrus A3: Estrus A4: Metestrus

In the control and α-lipoic acid groups, all animals (100%) exhibited a regular estrous cycle lasting 4–5 days, including the proestrus, estrus, metestrus, and diestrus phases. In the Hp group, 100% of the animals showed a prolonged diestrus phase starting from the 7th day after PTU administration, and the normal cyclic pattern was disrupted. Estrus was absent in most animals after approximately day 14. In the Hp + α-lipoic acid group, treatment partially restored the estrous cyclicity. Estrus findings were observed in 57% of the animals after 7 days of treatment and in 100% of the animals after 14 days of treatment, indicating recovery of the cycle. On the final day of the experiment, the distribution of estrous phases in the Hp + α-lipoic acid group was closer to that of the control group. These findings suggest that α-lipoic acid administration ameliorated the Hp-induced disruption of the estrous cycle (Fig. 4).

**Fig. 5:**
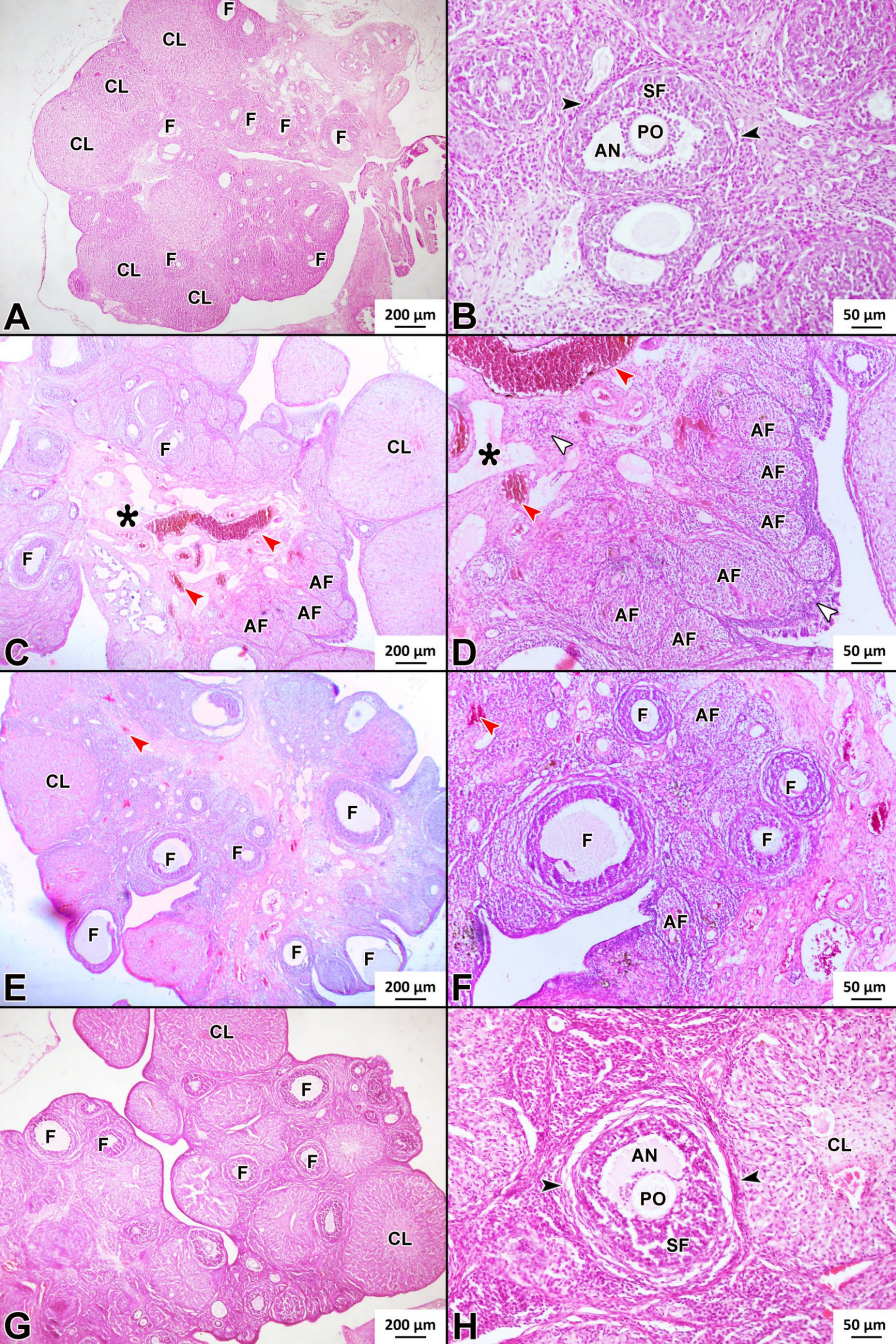
H&E staining of control (**A** and **B**), Hp (**C** and **D**), Hp + α-lipoic acid (**E** and **F**), α-lipoic acid (**G** and **H**) groups. Developing follicles (F) with normal histology, corpus luteum (CL), primary oocyte with normal morphology (PO), antrum (AN), and a secondary follicle (SF) containing a theca layer (black arrowhead) in the control and α-lipoic acid groups. In the Hp group, decreased developing follicles (F) and corpus luteum (CL), numerous atretic follicles (AF), edema (asterisk), inflammatory cell infiltration (white arrowhead), and vascular congestion (red arrowhead) are observed. In the Hp + α-lipoic acid group, increased developing follicles (F) and corpus luteum (CL), and reduced atretic follicles (AF) and congestion (red arrowhead) are seen compared with the Hp group. Magnification **A**, **C**, **E** and **G** 40X; **B**, **D**, **F** and **H** 200X

Hematoxylin and eosin (H&E) staining was performed on ovarian tissues from all groups, and semi-quantitative scoring was used for histological evaluation (Fig. 5). In the control (Fig. 5A and B) and α-lipoic acid (Fig. 5G and H) groups, ovarian tissues displayed normal histological appearance with typical ovarian morphology, including all follicle types at different stages of maturation as well as the corpus luteum, an indicator of ovulation. Granulosa and theca cells showed a regular and intact structure, and no degeneration was observed in the oocyte or surrounding zona pellucida. Semi-quantitative scoring revealed no statistically significant differences in hemorrhage (*p* = 1.00), follicular cell degeneration (*p* = 1.00), inflammatory cell infiltration (*p* = 1.00), or edema (*p* = 1.00) between the control and α-lipoic acid groups. In the Hp group (Fig. 5C and D), when compared with the control group, ovarian morphology showed disrupted structure: granulosa cell layers were reduced and loosely arranged in many follicles. Apoptotic granulosa cells with pyknotic nuclei, indicative of follicular atresia, were especially evident in secondary follicles. Semi-quantitative scoring demonstrated that hemorrhage (*p* < 0.001), follicular degeneration (*p* < 0.001), inflammatory cell infiltration (*p* < 0.001), and edema (*p* < 0.001) were significantly increased compared with the control group. In contrast, the Hp + α-lipoic acid group (Fig. 5E and F) showed ovarian histology closer to normal. Follicular degeneration, leukocyte infiltration, edema, and vascular congestion were significantly reduced compared with the Hp group (*p* < 0.05), indicating that α-lipoic acid treatment ameliorated histopathological damage (Table 2).

**Table 2:** Semi-quantitative score analysis of histopathological results in rat ovarian tissues from the experimental groups. Values are presented as median (min–max). Statistical analysis was performed using the Kruskal–Wallis test followed by Dunn’s post hoc test. a: Compared to the control group, *p* < 0.05; b: Compared to the Hp group, *p* < 0.05; c: Compared to the Hp + α-lipoic acid group, *p* < 0.05

Groups	Follicular Degeneration Median (Min–Max)	Inflammatory Cell Median (Min–Max)	Edema Median (Min–Max)	Vascular Congestion Median(Min–Max)	*p* values
Control Group	0.00 (0.00–0.10)^a^	0.00 (0.00–0.20)^a^	0.00 (0.00–0.10)^a^	0.00 (0.00–1.20)^a^	*p* < 0.05
Hp Group	2.00 (2.00–3.00)^b^	1.00 (0.00–1.00)^b^	3.00 (2.00–3.00)^b^	2.00 (2.00–3.00)^b^	
Hp + α-Lipoic acid Group	1.00 (0.00–1.00)^c^	1.00 (0.00–1.00)^c^	1.00 (0.00–1.00)^c^	1.00 (0.00–1.00)^c^	
α-Lipoic acid Group	0.00 (0.00–0.10)^a^	0.00 (0,00–0,20)^a^	0.00 (0.00–0.10)^a^	0.00 (0.00–1.20)^a^	

#### GRP78, PI3K, and AKT1 tissue expression results

**Fig. 6:**
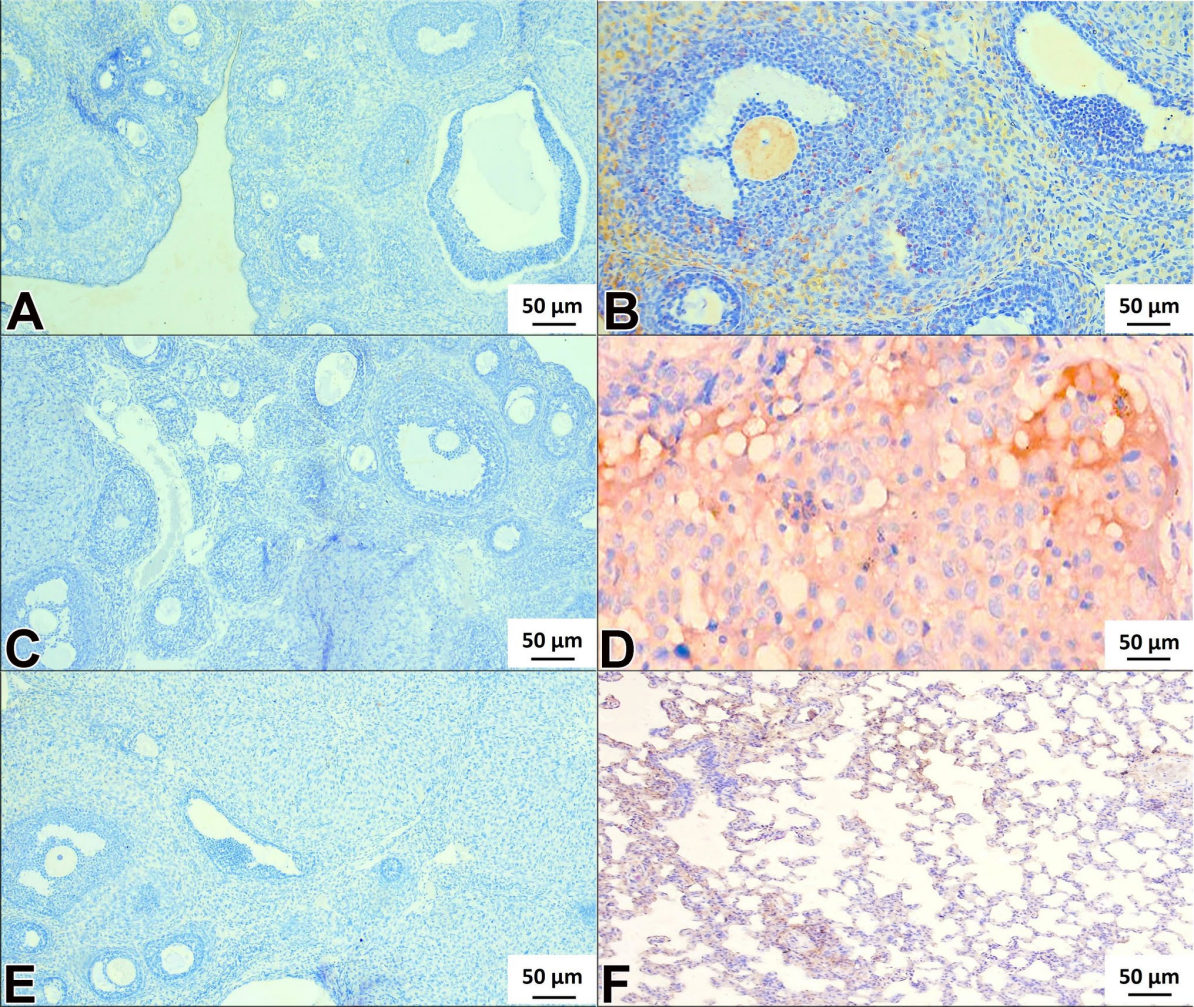
GRP78, AKT1, and PI3K immunoreactivity in positive and negative control tissues used for immunohistochemical staining. (**A**: GRP78 negative control ovarian tissue, **B**: positive control ovarian tissue, **C**: PI3K negative control ovarian tissue, **D**: Human lung cancer positive control, **E**: AKT1 negative control ovarian tissue, positive control: rat lung tissue AEC chromogen, Mayer's hematoxylin. Scale bar: **A**, **C**, **E** anf **F** 50 µm, **B** 100 µm, **D** 200 µm

**Fig. 7:**
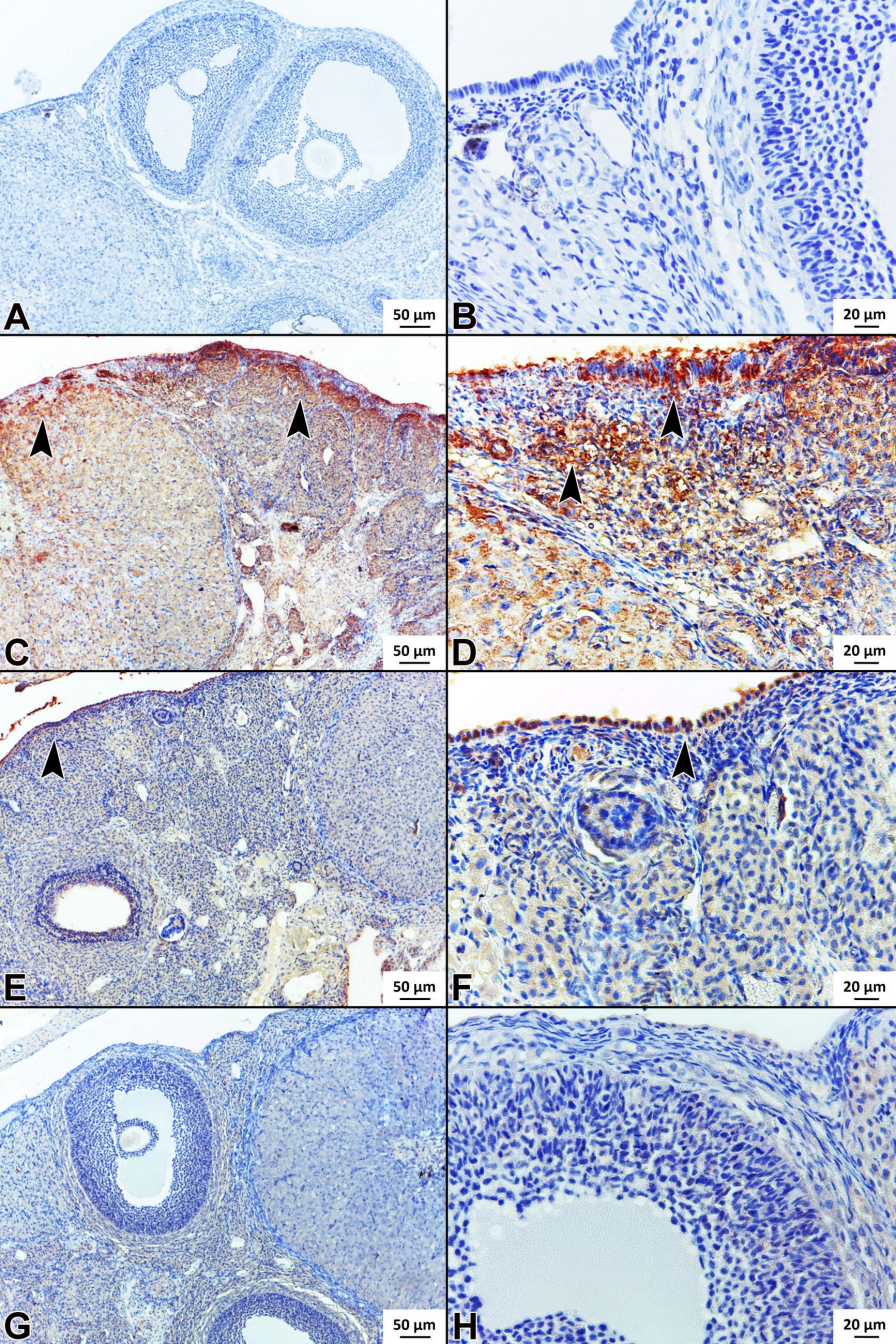
GRP78 immunostaining of control (**A** and **B**), Hp (**C** and **D**), Hp + α-lipoic acid (**E** and **F**), α-lipoic acid (**G** and **H**) groups. Positive immunostaining (arrowhead). Magnification **A**, **C**, **E** and **G** 200X; **B**, **D**, **F** and **H** 400X

**Fig. 8:**
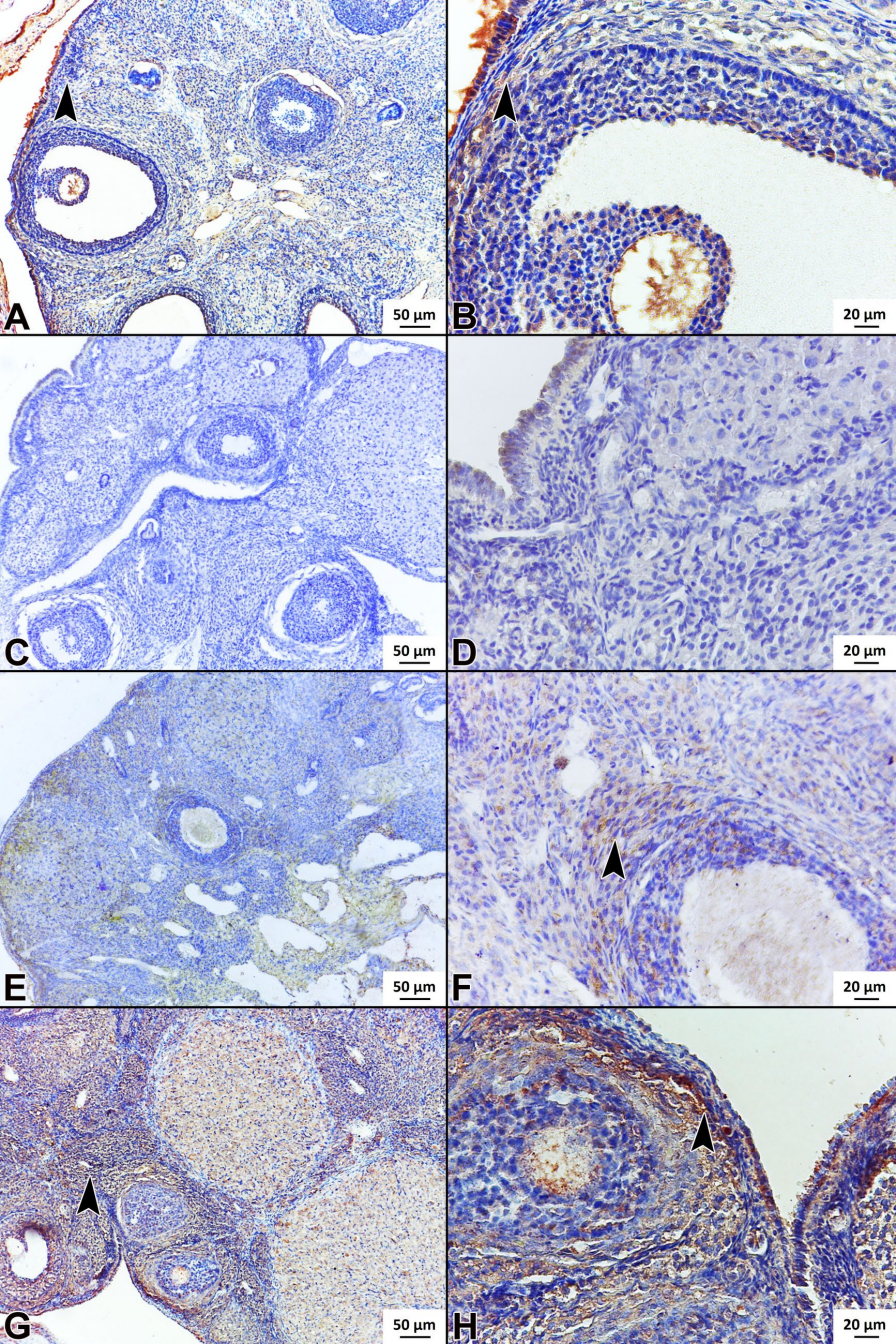
PI3K immunostaining of control (**A** and **B**), Hp (**C** and **D**), Hp + α-lipoic acid (**E** and **F**), α-lipoic acid (**G** and **H**) groups. Positive immunostaining (arrowhead). Magnification **A**, **C**, **E** and **G** 200X; **B**, **D**, **F** and **H** 400X

**Fig. 9:**
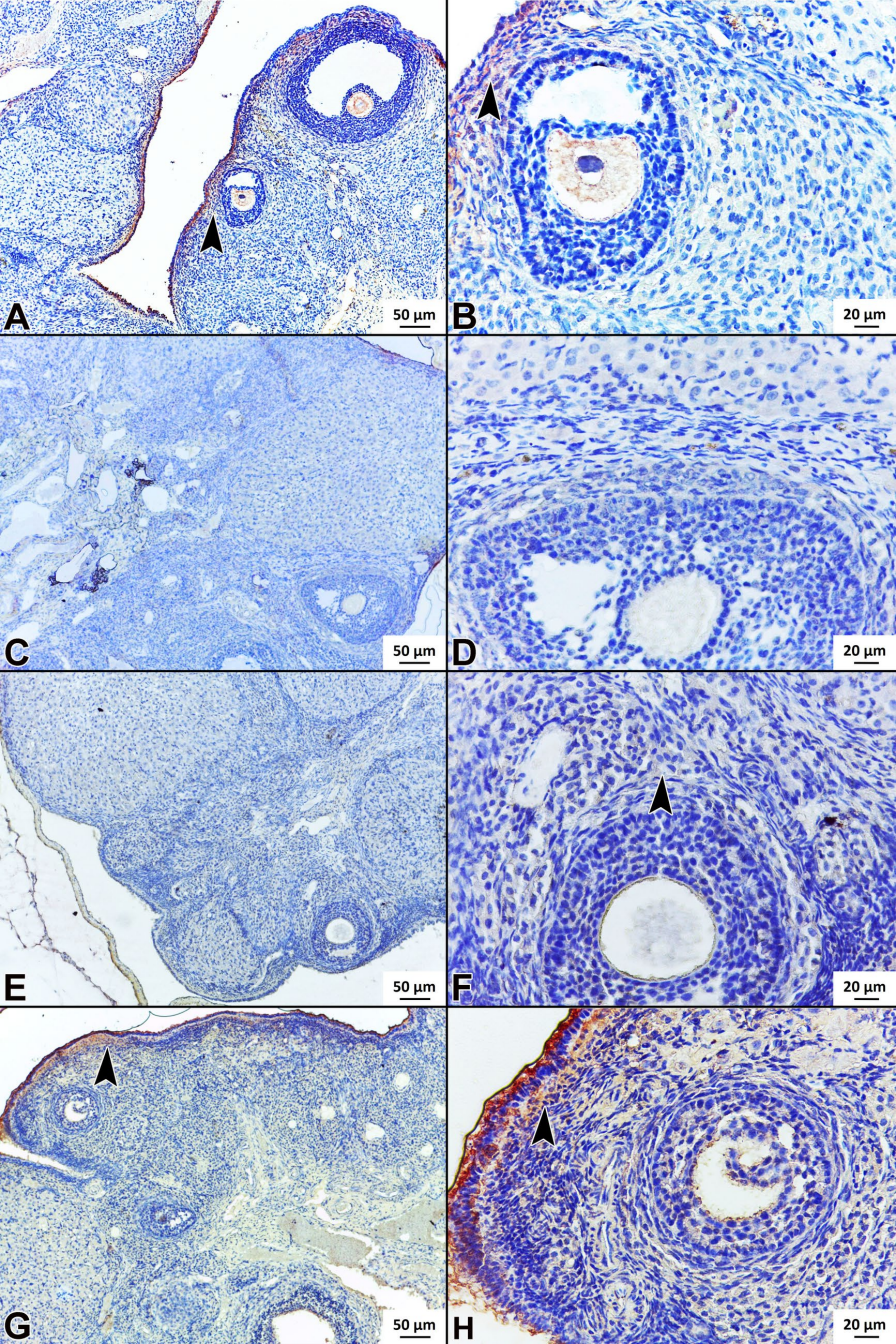
AKT1 immunostaining of control (**A** and **B**), Hp (**C** and **D**), Hp + α-lipoic acid (**E** and **F**), α-lipoic acid (**G** and **H**) groups. Positive immunostaining (arrowhead). Magnification **A**, **C**, **E** and **G** 200X; **B**, **D**, **F** and **H** 400X

**Fig. 10:**
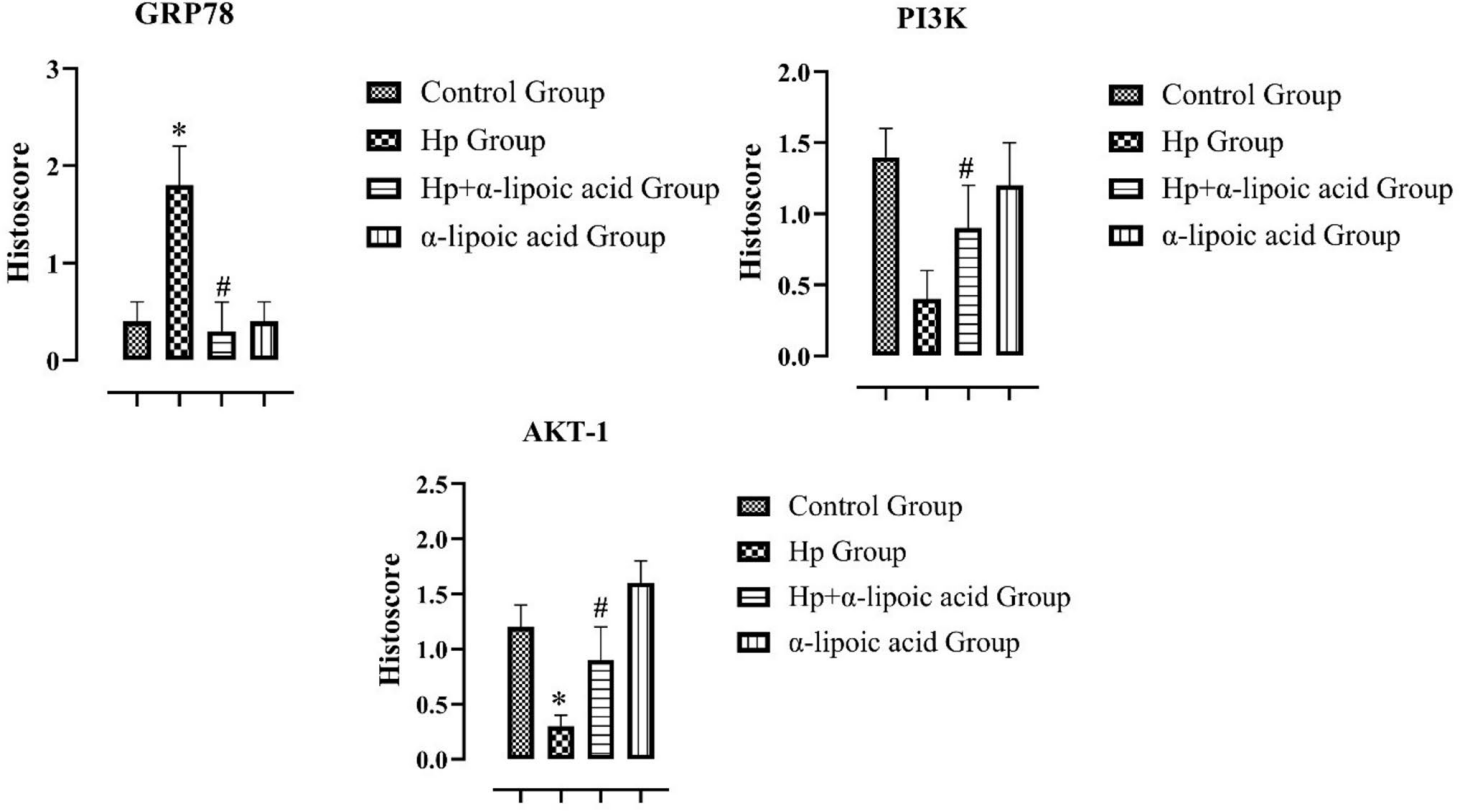
Immunoreactivity levels of GRP78, PI3K, and AKT1 in rat ovarian tissues from the experimental groups. Statistical analysis was performed using the Kruskal–Wallis test followed by Dunn’s post hoc test. Data were analyzed as median (min–max). * Compared to the control group, *p* < 0.05; # Compared to the Hp group, *p* < 0.05)

Positive and negative controls for GRP78, PI3K, and AKT1 were performed in the ovarian tissues of all groups (Fig. 6). Subsequently, the levels of GRP78 (Fig. 7), PI3K (Fig. 8), and AKT1 (Fig. 9) in the ovarian tissues of all groups were evaluated. In the control and α-lipoic acid groups, GRP78 (*p* = 0.428), PI3K (*p* = 0.920), and AKT1 (*p* = 0.326) levels were comparable. Compared with the control group, the Hp group showed significantly increased tissue GRP78 levels (*p* < 0.001), whereas PI3K and AKT1 levels were significantly decreased (*p* < 0.001). In the Hp + α-lipoic acid group, GRP78 expression was significantly decreased (*p* < 0.001), while PI3K and AKT1 levels were significantly increased (*p* < 0.001) compared with the Hp group (Fig. 10).

## Discussion

The findings obtained in this study revealed that significant biochemical and histopathological changes occurred at both serum and ovarian tissue levels in the hypothyroidism model created with propylthiouracil (PTU). PTU administration resulted in a significant decrease in serum T3, T4 and LH levels and a marked increase in TSH, and FSH levels. Furthermore, oxidative stress was evident, as indicated by decreased TAS and elevated TOS levels, while GRP78 expression was increased and PI3K and AKT1 levels were reduced. Histological examinations revealed structural abnormalities such as a decrease in follicle numbers, follicular degeneration, inflammatory cell infiltration, and edema. However, α-lipoic acid treatment significantly improved these biochemical and histopathological changes associated with hypothyroidism, showing protective effects, particularly in oxidative stress, folliculogenesis, and PI3K/AKT1 signaling pathways. These results suggest that α-lipoic acid may be a potential therapeutic agent against hypothyroidism-related ovarian damage.

Several studies have reported that PTU, through negative feedback on the hypothalamic-pituitary-thyroid axis, increases TSH release from the pituitary gland while reducing serum T3 and T4 levels, thus serving as a reliable marker in hypothyroidism models (McDermott 2019; Hershman and Beck-Peccoz 2023). In line with these reports, the present study found that PTU-treated rats exhibited significantly elevated TSH and decreased T3, T4 levels. There are conflicting reports regarding FSH and LH levels in rats treated with PTU. In a study involving 8 weeks of PTU treatment FSH and LH levels increased significantly in the Hp group compared to the control group (Ezzat et al. 2024) whereas LH levels decreased after 60 days of PTU treatment. (Santos et al. 2023). In another 8-week PTU study, FSH and LH levels decreased in the Hp group compared to the control group (El-Kashlan et al. 2015). In our study, FSH decreased and LH decreased in the HP group compared to the control group. In female ovarian damage models, ALA normalizes elevated FSH (Nair et al. 2020) and LH levels (Nair et al. 2020; Özel et al. 2020). In our study, the absence of changes in hormone panels (TSH, T3, T4, FSH, and LH) in the Hp group treated with ALA compared to the Hp group demonstrated that ALA does not directly affect hormones in hypothyroidism. Rat and Human thyroid data are limited (Khoder et al. 2022; Tripathi et al. 2023).Thus, ALA administration in rats with PTU-induced hypothyroidism may have been insufficient to alter hormones in terms of duration and dose.

Redox imbalance has been increasingly recognized as a key mechanism in the etiology and pathophysiology of thyroid diseases and in other complex metabolic disorders (Wang et al. 2021). Previous studies have consistently shown increased oxidative stress during both subclinical and overt hypothyroidism, with reduced TAS and elevated TOS levels (Nanda et al. 2008). In PTU-induced hypothyroidism, both thyroid hormone deficiency and PTU-related oxidative tissue damage contribute to the depletion of total antioxidant defense (Bozbek and Şentürk 2023). The present study similarly revealed reduced TAS and elevated TOS levels in the hypothyroid group. α-lipoic acid has been reported to function not only as a strong antioxidant but also as a cofactor for various enzymatic complexes such as pyruvate dehydrogenase, thereby supporting energy metabolism and cell survival (Maglione et al. 2015). Studies on animals and in vitro demonstrate that ALA lowers oxidative indicators and enhances antioxidant systems in the brain, liver, ovary, and heart, frequently returning TAS/TOS-like indices to normal (Calabrese et al. 2023). In our study, alpha-lipoic acid administered systemically exerted antioxidant effects by increasing total antioxidant status (TAS) and reducing total oxidant status (TOS).

Prolonged alterations in TSH concentration have been associated with a broad range of reproductive impairments, including disrupted folliculogenesis, impaired ovulation and fertilization rates, and, in severe cases, ovarian failure (Vissenberg et al. 2015). Although a reduction in thyroid hormones is generally accepted to influence ovarian folliculogenesis, the precise mechanisms of chronic hypothyroidism on follicular development and ovarian reserve remain poorly defined (Meng et al. 2017). These hormonal changes may contribute to follicular degeneration and an increased number of atretic follicles (Paiva et al. 2024), and also impact tertiary follicle and corpus luteum counts (Armada-Dias et al. 2001). In particular, reduced T3 and T4 levels fail to act synergistically with FSH to stimulate granulosa cell development, resulting in decreased primary follicle numbers (de Albuquerque et al. 2020; Das et al. 2023). In a study evaluating the effects of chronic hypothyroidism developing in adulthood on ovarian reserve and folliculogenesis, hypothyroid rats showed a significant decrease in the numbers of primordial, primary, and preantral follicles, along with a trend toward reduced numbers of antral follicles and corpora lutea (Meng et al. 2017). Similarly, in our study, compared with the control group, the Hp group showed a significant decrease in the numbers of healthy follicles (primordial, primary, secondary, and tertiary) and corpora lutea, while the number of atretic follicles increased. ALA decreases apoptosis and fibrosis in ovaries exposed to cryodamage, chemotherapy, toxins, or metabolic stress, helping maintain primordial and growing follicles (Soylu Karapinar et al. 2017; Özel et al. 2020; Colcimen and Keskin 2025). Histological findings showing increased numbers of primordial, primary, secondary, and tertiary follicles and corpora lutea, together with a reduction in atretic follicles following α-lipoic acid treatment, suggest that α-lipoic acid mitigates hypothyroidism-induced ovarian damage and partially improves ovarian reserve.

Glucose-regulated protein 78 (GRP78) is an endoplasmic reticulum (ER) stress marker involved in cell survival and expressed in various tissues (Liu et al. 2023). Previous studies have reported that GRP78 protein levels are significantly decreased in both hypo- and hyperthyroid rats compared with controls, indicating that hypothyroidism and thyroid hormone imbalance disrupt GRP78-mediated endoplasmic reticulum stress pathways in the ovaries (Wang et al. 2020; Liu et al. 2023). In the present study, GRP78 levels were significantly elevated in the Hp group compared to control, while α-lipoic acid treatment (Hp + α-lipoic acid group) significantly reduced ovary GRP78 levels. Although previous studies have reported reduced GRP78 levels under thyroid hormone imbalance (Liu et al. 2025), the increased GRP78 observed in the Hp group in the present study may reflect a compensatory upregulation in response to enhanced oxidative and ER stress. This discrepancy may be attributed to differences in experimental models and tissue-specific responses. In line with our findings, a study demonstrating that heat stress increases endoplasmic reticulum stress and upregulates GRP78 expression in ovarian tissue reported that ALA significantly suppressed the heat stress–induced increase in GRP78 levels in ovarian tissues (Xiong et al. 2021).

PI3K/Akt signaling play roles for oogenesis, folliculogenesis, oocyte maturation and early embryo development; dysregulation leads to follicle atresia and infertility (Yao et al. 2018). Thyroid hormones also influence ovarian function (Silva et al. 2018). Although the roles of the PI3K/Akt signaling pathway in the ovary have been extensively investigated in conditions such as ovarian cancer, polycystic ovary syndrome (PCOS), primary ovarian insufficiency, and premature ovarian failure (De Felici and Klinger 2021; Hu et al. 2025), direct mechanistic data linking this pathway to ovarian tissue in hypothyroid women remain limited. Nevertheless, the available evidence suggests that hypothyroidism may contribute to impaired folliculogenesis and reduced fertility by downregulating PI3K/Akt signaling in granulosa cells (Vissenberg et al. 2015). These findings, together with previous literature, suggest that oxidative stress induced by hypothyroidism contributes to ovarian histopathological damage, while α-lipoic acid ameliorates tissue injury, enhances antioxidant capacity, preserves ovarian reserve, and restores PI3K/AKT signaling.

This study has several limitations. First, propylthiouracil-induced hypothyroidism was conducted solely in a rat model, limiting direct extrapolation to human physiology. Second, only one dose of α-lipoic acid (100 mg/kg/day) was administered, and alternative dosing regimens or treatment durations were not explored. Measurement of serum reproductive hormones such as progesterone, FSH, LH, estradiol, and estrogen would have provided functional endocrine correlates to the observed histological and molecular changes. The lack of these hormonal assessments therefore represents a limitation, as they could have strengthened the translational and clinical relevance of the study. While biochemical and histological parameters were evaluated, molecular mechanisms were not fully investigated; analyses such as gene expression profiling or proteomic studies were not performed. In conclusion, further studies are needed to elucidate the role of GRP78/PI3K/AKT signaling in ovarian damage caused by hypothyroidism. Additionally, α-lipoic acid, as a promising supportive agent, warrants further investigation in combination with other treatments for ovarian injury.

### Clinical implications

The clinical relevance of the present study lies in its potential implications for women with hypothyroidism, particularly those of reproductive age who are at increased risk of impaired folliculogenesis and reduced ovarian reserve. Our findings suggest that hypothyroidism-induced ovarian damage may be mediated through oxidative stress, ER stress (GRP78), and suppression of the PI3K/Akt signaling pathway. Although further clinical studies are needed, our results provide a translational basis for considering antioxidant-based interventions in hypothyroid women with reproductive dysfunction.

## Data Availability

All source data for this work (or generated in this study) are available upon reasonable request.

## Declarations

This study was conducted in accordance with the principles of the Declaration of guide by *NIH*. This study received ethical approval from the Adıyaman University Local Ethics Committee for Laboratory Animals on November 24, 2022 (Approval number: 2022/068).
